# Birth and prenatal care outcomes of Latina mothers in the Trump era: Analysis by nativity and country/region of origin

**DOI:** 10.1371/journal.pone.0281803

**Published:** 2023-03-01

**Authors:** Carmen Gutierrez, Nathan T. Dollar

**Affiliations:** 1 Department of Public Policy, Carolina Population Center, University of North Carolina at Chapel Hill, Chapel Hill, North Carolina, United States of America; 2 Carolina Population Center, University of North Carolina at Chapel Hill, Chapel Hill, North Carolina, United States of America; University of Notre Dame Keough School of Global Affairs, UNITED STATES

## Abstract

We examined whether and how birth outcomes and prenatal care utilization among Latina mothers changed over time across years associated with the Trump sociopolitical environment, using restricted-use birth records from the National Center for Health Statistics (NCHS). To assess potential variation among subpopulations, we disaggregated the analyses by maternal nativity and country/region of origin. Our results indicate that both US- and foreign-born Latina mothers experienced increasingly higher risks of delivering low birthweight (LBW) and preterm birth (PTB) infants over the years associated with Trump’s political career. Among foreign-born Latinas, adverse birth outcomes increased significantly among mothers from Mexico and Central America but not among mothers from Puerto Rico, Cuba, and South America. Levels of inadequate prenatal care utilization remained largely unchanged among groups who saw increases in LBW and PTB, suggesting that changes in prenatal care did not generally explain the observed worsening of birth outcomes among Latina mothers during the Trump era. Results from this study draw attention to the possibility that the Trump era may have represented a source of chronic stress among the Latinx population in the US and add to the growing body of literature linking racism and xenophobia in the sociopolitical environment to declines in health among Latinx people, especially among targeted groups from Mexico and Central America.

## Introduction

A growing body of evidence suggests that the political career of Donald Trump has generated harmful consequences for the health and wellbeing of Latinx people in the United States. Throughout his 2016 presidential campaign and subsequent presidency, Trump prioritized an immigration platform that included explicit and implicit anti-immigrant and anti-Latinx policies and rhetoric [1,2]. Several studies have since linked Trump’s racist rhetoric and political agenda to increased levels of psychosocial stress and anxiety among the Latinx population [3–9]. This sociopolitical stress has been linked to declines in health among Latinx adults and children, and there are important reasons to expect that it may produce particularly damaging and long-term consequences for society’s next generation: newborn infants.

Sociopolitical stressors during pregnancy have been linked to adverse birth outcomes, including low birthweight (LBW) and preterm birth (PTB) [10–15], which have far-reaching health effects, such as problems with cognitive development and chronic diseases in adulthood [16,17]. Maternal stress can affect birth outcomes through several complex biological pathways, including the mother’s physiological, immune, and neuroendocrine responses to stress which can harm the developing fetus [18,19]. Birth outcomes can also be affected by negative changes in health-promoting behaviors, such as lowered utilization of prenatal care [18,20], which may be a particular concern among Latina mothers who face unique discrimination and threats by the US healthcare system [21,22].

Several studies have documented adverse birth outcomes among Latina mothers after discrete anti-immigrant events. One study found a 24% greater risk in LBW among births to both US- and foreign-born Latina mothers in Postville, Iowa following a federal immigration raid that took place there in 2008, compared with births prior to the raid [12]. Another study found that the introduction of the 287(g) program, which grants local police the authority to enforce federal immigration responsibilities, was associated with reductions in birthweight and worsening in the use of prenatal care among foreign-born mothers in the Mecklenburg County of North Carolina where Latina immigrants made up approximately 95% of the sample [23]. The passage of Arizona’s SB1070 “show me your papers” law was similarly associated with a significant decline in birthweight among births to Latina immigrant women, even in the absence of actual enforcement of the law [24].

Two recent studies have investigated potential changes in birth outcomes among Latina mothers as a result of anti-immigrant policy and rhetoric in the Trump sociopolitical environment, specifically. One study compared the risk of PTB between periods before and after Trump’s presidential inauguration using birth records in New York City and found a significant increase in the rate of PTB among Latina mothers, with the greatest increase among foreign-born Latinas [14]. In the first national-level study of this type, Gemmill and colleagues showed that Trump’s 2016 presidential election was associated with a 3.2% to 3.6% increase in PTB among infants born to Latina mothers [13]. Unlike the previous study, the researchers were not able to disaggregate their results by nativity or country/region of origin.

While these studies provide valuable contributions to our understanding of how birth outcomes among Latina mothers in the US are affected by structural racism, in general, and by Trump’s political career, specifically, key gaps in our knowledge remain. First, surprisingly little work has considered how racist and anti-immigrant sociopolitical events serve as chronic sources of stress. Discrete events can be linked to specific dates with accuracy, and researchers tend to measure potential effects of stress in a period of a few weeks or months after an event. Stress caused by a discrete event, however, likely persists well beyond a few weeks or months. Indeed, racist and anti-immigrant events have the capacity to fully reshape the sociopolitical environment, creating a chronic experience of stress for long periods of time [8].

Second, most studies in this area have focused on birth outcomes among women who are encompassed by the broad umbrella terms of “Hispanic” or “Latina.” In terms of research in the Trump era specifically, no study has examined how national-level changes in birth outcomes among infants born to Latina mothers differ by nativity or country/region of origin. There are important reasons to expect, however, that the health effects of racist and xenophobic policies and rhetoric would vary within the Latinx population [25–27]. People who experience greater fears about deportation or family separation, for example, may experience worse health outcomes due to suffering higher levels of stress and anxiety. Such fears are not equally distributed among Latinx people because the likelihood of being undocumented, or having an undocumented family member, is differentially concentrated, with higher levels among those from Mexico and Central America and lower levels among people from Cuba and South America [28]. People may also experience worse health as a result of being suspected as undocumented, regardless of their citizenship status [29]. This experience further contributes to health differences within the Latinx population, given that most people link “illegality” to individuals who are Mexican [30]. Thus, certain groups of Latin American mothers, such as those from Mexico and Central America, may have experienced worse birth outcomes during Trump’s political career due to the greater fears, anxieties, and vulnerabilities that come along with being undocumented or simply being suspected as undocumented.

Third, more attention is needed on the potential mechanisms that could explain the observed link between sociopolitical stress and adverse birth outcomes among Latina mothers, such as changes in the use of prenatal care. Existing literature finds that racist, anti-immigrant events can lead to “chilling effects,” whereby feelings of fear, anxiety, and mistrust prevent immigrants from seeking needed healthcare services [31–35]. A recent study of patients in Houston, Texas found significant declines in prenatal care visits among expectant immigrant Latina mothers from Mexico and Central American following the launching of Trump’s campaign in the summer of 2015 [21]. Whether and how prenatal care utilization in the Trump era has changed among Latina women at the national level, however, remains unknown.

To address these important and timely gaps in the literature, we examined the associations between the Trump sociopolitical environment and birth outcomes and prenatal care utilization among Latina women over a six-year period. While these associations are temporal and not causal, our analyses nevertheless allow us to document whether and how racist, anti-immigrant events associated with the Trump sociopolitical environment may have represented chronic sources of stress for the Latinx population in the United States. To assess potential heterogeneity within the Latina immigrant population, we leveraged information in the restricted-use birth records from the National Center for Health Statistics (NCHS) that allowed us to disaggregate results by maternal country/region of origin. Given Trump’s specific targeting of and scrutiny against people from Mexico and Central America [1–7], we hypothesized that immigrants from these countries would experience more severe negative changes to their birth outcomes and prenatal care utilization, relative to foreign-born Latinas from other countries and US-born Latinas.

## Methods

### Study design

We conceptualized the entirety of Trump’s presidential campaign and subsequent administration as a period of chronic stress for Latinx people in the United States. At the same time, we also expected that stress levels affected by this sociopolitical environment may have varied over time as Trump’s racist, anti-immigrant rhetoric and policy agenda became increasingly threatening over the course of his political career. Accordingly, we designed this study to compare birth outcomes and levels of prenatal care across each year of Trump’s political career, including his presidential campaign, election, and administration, relative to a “pre-Trump” period. Descriptions of these periods are provided in Table 1.

**Table 1:** Description of study periods in relation to the 2016 U.S. presidential election.

Period	Dates	Context
2014 Pre-Trump Period (Comparison Period)	July 1 –Dec 31 2014	Pre-Trump Period
2015 Post-Trump Period	July 1 –Dec 31 2015	Donald Trump announces candidacy for U.S. president(June 16) • During his campaign announcement speech, Trump refers to Mexican immigrants as “criminals” and “rapists” and promises to build a “great wall” along the US-Mexico border • Trump’s campaign speech also mentions anti-Latinx rhetoric, more broadly, stating that “It’s coming from more than Mexico. It’s coming from all over South and Latin America…” • Trump’s negative speech toward Mexicans and other Latinx people were prominent in his speeches, interviews, and on his Twitter account throughout his campaign • Trump regularly retweeted messages from white supremacists and neo-Nazis through his campaign
2016 Post-Trump	July 1 –Dec 31 2016	Donald Trump accepts the republican nomination (July 21)/Wins U.S. presidential election (Nov 8) • In victory speech, Trump stated that “countries like Mexico are…killing us on the border…destroying us in terms of economic development” and once again discussed his promise to build a wall along the US-Mexico border • Trump’s level of negative tweets mentioning Mexicans and Latinx people reached its highest level during the week of the 2016 US presidential election • Trump released a video message summarizing six priorities for his administration in its first 100 days in which he offered 10 policy actions he would take to address immigration reform, including the following items: having zero tolerance for criminals who live in the US illegally; blocking funding for sanctuary cities; and ending employment and benefits for individuals residing in the country without legal permission • Trump publicly remarks that US District Judge, Gonzalo Curiel hearing the case against Trump University may be biased because of his Mexican heritage
2017 Post-Trump	July 1 –Dec 31 2017	Donald Trump Inaugurated as U.S. President (Jan 20) • Trump issues Executive Order 13788 “Enhancing Public Safety in the Interior of the United States,” to hire 10,000 additional immigration officers, deputize state and local law officials to perform immigration enforcement, penalize sanctuary city jurisdictions, and reinstate the Secure Communities program • Trump issues Executive Order 13767 “Border Security and Immigration Enforcement Improvements,” to secure the US-Mexico border through the construction of a physical wall and the expansion of detention capacity and authority along the border • Trump administration releases the 2018 fiscal year budget requesting for 1.5 billion dollars to add over 50,000 beds to immigration detention facilities • Under Trump’s direction, Immigration and Customs Enforcement (ICE) publicly acknowledge it has adopted a new policy called the Surge Initiative, in which parents or sponsors of unaccompanied minors are detained or subjected to enforcement actions • Fulfilling one of Trump’s key campaign promises, Attorney General Jeff Sessions rescinded the Deferred Action for Childhood Arrivals (DACA) program
2018 Post-Trump	July 1 –Dec 31 2018	Donald Trump’s 2^nd^ year in Office • Under Trump the Department of Justice sues California for “sanctuary” state policies’ • Trump warns of dangerous “caravans” of immigrants headed to the US border from Central America • Trump directs U.S. Attorneys Offices along US-Mexico border to prosecute all DHS referrals of illegal entry and attempted illegal entry • Under Trump, Attorney General Jeff Sessions decides matter that makes it more difficult to obtain protection for asylum and refugee seekers feeling domestic violence or gang violence • Trump issues Executive Order 13841 “Affording Congress the Opportunity to Address Family Separation,” which reiterates his commitment to rigorously enforce our immigration laws and replaces family separation policy with policy of prolonged family detention
2019 Post-Trump	July 1 –Dec 31 2019	Donald Trump’s 3^rd^ year in Office • Trump administration announces that 3,700 more troops would be sent to the US-Mexico border to assist Customs and Border Protection by placing wire along the border and helping with surveillance operations • Trump signs a $328 billion spending bill that included $1.375 billion in funding for barriers on the US-Mexico border, and declared a state of emergency on the US-Mexico border because he did not receive the $5.7 billion in funding that he had requested for the border wall • Trump issues Executive Order 13880 “Collecting Information About Citizenship Status in Connection with the Decennial Census,” to include a question inquiring about citizenship status on the decennial census, which was seen as a specific attempt to instill fear among undocumented Latinx people • During a campaign rally, when a supporter shouted “shoot them” in reference to undocumented immigrants, Trump responds by saying “only in the Panhandle”

We considered the formal launching of Trump’s presidential campaign as the primary marker of time when we might expect to observe negative changes in adverse birth outcomes and prenatal care utilization among Latina mothers, owing to experiences of racism and discrimination during pregnancy. In the June 2015 speech announcing his candidacy, Trump established the racist and anti-immigrant tone of his campaign by referring to Mexican immigrants as “criminals” and “rapists” and promising to build a “great wall” along the US-Mexico border. His campaign speech also went on to disparage all Latinx people, stating that crime is “…probably coming from more than Mexico. It’s coming from all over South and Latin America…”. Trump’s racist and xenophobic speech toward Mexicans and other Latinx groups continued in his speeches, interviews, and were prominent on his Twitter account throughout his campaign [1]. To capture the potential heightened stress associated with this overtly racist rhetoric and policy agenda, we selected the six-month period from July 1, 2015 through December 31, 2015 as the first treatment period. For purposes of consistency and to address issues of seasonality associated with birth outcomes [36,37], we used the same six months in each subsequent year of our data to construct the additional treatment periods. Each successive period captures the emergence of Trump as a legitimate political figure and corresponds well with an escalation of racial hostility and anti-immigrant political rhetoric and policy implementation (see Table 1).

### Data

Our analyses are based on restricted-use natality data for the years 2014–2019, collected by the National Center for Health Statistics (NCHS) as part of the National Vital Statistics System [38–41]. We received permission to use the restricted natality files form the NCHS and the National Association for Public Health Statistics and Information Systems (NAPHSIS). Unlike the public-use data, these files contain information on maternal country of origin, allowing us to disaggregate births among immigrant Latina mothers and identify groups that have been disproportionately targeted by Trump, including populations from Mexico and Central America [1–7]. Altogether, our analysis allows us to identify mothers born in the following countries/regions: Mexico, Central America, South America, Puerto Rico, and Cuba. Important to note is that, unlike the other groups mentioned here, people born in Puerto Rico are US citizens. Still, people born in Puerto Rico have different lived experiences from Puerto Ricans born in the US, and birth outcomes between these groups are also known to be different [42,43]. To recognize these distinctions, we still refer to people born in Puerto Rico as “foreign-born” but acknowledge that this label is flawed.

In line with our study design, we limited our analytic dataset to include only births that occurred from July 1^st^ through December 31^st^ of each study year. Among these cases, we restricted our analysis to singleton live-births to Latina mothers who were residents of one of the 50 US states and who had complete information on maternal country/region of birth, age, formal schooling, parity, infant birthweight, and infant gestational age. Cases removed due to missing information made up less than 1% of all birth records in our study years.

The Institutional Review Board of the University of North Carolina at Chapel Hill approved the study protocol. All data was fully anonymized before we accessed them. All analyses and reporting of results were conducted in accordance with our data use agreement with NCHS/NAPHSIS.

### Measures

We examined two sets of outcomes: infant health at birth and maternal use of prenatal care. The infant health outcomes we considered were low birthweight (LBW; < 2,500 grams) and preterm birth (PTB; < 37 weeks gestation) [44]. Maternal use of prenatal care was assessed using Kotelchuck’s (1994) Adequacy of Prenatal Care Utilization Index (APNCU) [45]. This APNCU index has been previously validated as the “gold standard” for assessing adequacy of prenatal care use because of its incorporation of the month that prenatal care began, the proportion of the number of visits recommended by the American College of Obstetrician Gynecologists, and the gestational age at delivery [46,47]. Like other recent studies in this area [21,23], we used the APNCU categories to create a dichotomous variable comparing mothers who had “inadequate” prenatal care to those who had higher levels of prenatal care. Inadequate prenatal care is defined as prenatal care begun after the fourth month or less than 50% of recommended prenatal visits received as stratified by gestational age at delivery.

To estimate exposure to the Trump sociopolitical environment, the key independent variable distinguished the pre- and post-Trump study periods (0 for the pre-Trump study period; 1, 2, 3, 4, 5 for the post-Trump study periods). The control variables included in the analysis were maternal age (<20, 20–24, 25–29, 30–35, 36–39, 40+ years), formal schooling (<high school, high school diploma, some college, college diploma+), and parity (first live birth or not).

### Statistical analysis

We performed a multistep analysis. First, we conducted preliminary and descriptive analyses to evaluate the distributions and temporal variability of study variables. In these analyses, we estimated univariate frequencies of the control variables (maternal age, formal schooling, and parity) as well as the outcome variables (LBW, PTB, and inadequate prenatal care utilization) as proportions across each of the study periods. We assessed whether the proportions in the post-Trump periods differed from the pre-Trump period using two-tailed *z*-tests.

Second, we estimated multivariable logistic regression models to identify the temporal associations between exposure to the Trump treatment periods and each outcome of interest: LBW, PTB, and inadequate prenatal care utilization. Rather than comparing outcomes to a reference group with the lowest risk of the outcome (e.g., white mothers), we stratified the models for each population group of interest: US-born Latinas, foreign-born Latinas (aggregated), foreign-born Mexicans, foreign-born Central Americans, foreign-born South Americans, foreign-born Puerto Ricans, and foreign-born Cubans. By analyzing within-group variation over time, each ethnic population in essence serves as its own control group, providing a clearer assessment of how the exposure-outcome association differs across Latina mothers by nativity and maternal country/region of birth [48]. All models controlled for maternal age, formal schooling, and parity. All model equations and full model results can be viewed in the S1 File (see S1 File). The main results from the key independent variable measuring the Trump study periods are the adjusted odds ratios of the outcome (LBW, PTB, and inadequate prenatal care utilization) during each of the post-Trump periods compared with the pre-Trump period.

Third, we tested the sensitivity of our results by accounting for potential sources of bias. One potential source of bias in our statistical models in the exclusion of maternal marital status, which is a well-documented predictor of infant birth outcomes and use of prenatal care [49–51]. Despite its important relationship with this study’s outcomes of interest, we excluded maternal marital status from our analyses due to significant changes in the reporting of this information during our study period. In 2017, California stopped providing record-level data on maternal marital status for births occurring in the state. For all birth records in the years 2017–2019, approximately 6% are missing maternal marital status. In our analytic sample of Latina mothers, approximately 12% are missing on maternal marital status between 2017 and 2019. To avoid biasing our results based on this missing data, our analyses exclude maternal marital status as a control variable. The exclusion of marital status from our analyses, however, may still influence our results due to omitted variable bias. To address this potential important source of bias in our analyses, we used multiple imputation to replace missing data on maternal marital status, and re-estimated our multivariable logistic regression models for each population group using the imputed data.

For all analyses, statistical significance was assessed at 2-sided *P* < .05. All analyses were conducted using Stata 15 SE software.

## Results

### Descriptive statistics and preliminary analyses

Table 2 shows the descriptive statistics for the maternal sociodemographic characteristics of the sample. Overall, the analytic sample consisted of 2,620,542 singleton live births, including 1,367,058 births to US-born Latina mothers and 1,253,484 births to foreign-born Latina mothers. Compared to US-born Latina mothers, foreign-born Latina mothers were generally younger, had fewer years of formal schooling, and higher levels of parity. Maternal age, schooling, and parity varied among foreign-born Latina mothers by country/region of origin (see Table 2). In terms of temporal variation, the results in Table 2 suggest that traditional risk factors associated with negative birth and prenatal care utilization outcomes were less prevalent in the post-Trump periods compared with the pre-Trump period. Across all groups, there were significant reductions (*P* < .05) in the rates of births by mothers who were less than 20 years old and who had less than a high school level of formal schooling.

**Table 2:** Descriptive statistics on maternal characteristics for US resident mothers across study periods (July 1 to Dec 31 in years 2014–2019).

	2014 Pre-Trump Period 2014	2015 Post-Trump Period 2015	2016 Post-Trump Period 2016	2017 Post-Trump Period 2017	2018 Post-Trump Period 2018	2019 Post-Trump Period 2019
**US-Born Latina** *N = 1*,*367*,*058*	*N = 217*,*810*	*N = 225*,*561*	*N = 229*,*168*	*N = 229*,*701*	*N = 230*,*483*	*N = 234*,*335*
Age						
< 20	13.4	12.2***	11.2***	10.6***	9.8***	9.3***
20–24	33.4	32.3***	31.2***	30.1***	28.8***	28.1***
25–29	26.6	28.1***	29.2***	30.1***	31.0***	31.4***
30–34	17.8	18.1**	18.5***	18.8***	19.6***	20.2***
35–39	7.3	7.8***	8.3***	8.7***	9.1***	9.1***
40+	1.4	1.5**	1.6***	1.7***	1.7***	1.8***
Formal Schooling						
< High School	19.9	18.7***	17.8***	16.7***	15.7***	14.7***
High School	33.3	33.3	33.7**	34.4***	34.5***	35.0***
Some College	33.9	34.4**	34.3**	34.4**	34.3*	34.2*
College+	12.9	13.6***	14.2***	14.7***	15.5***	16.0***
% First Birth	41.0	40.4***	40.0***	40.1***	40.2***	41.0
**Foreign-Born Latina**^**1**^ *N = 1*,*253*,*484*	*N = 208*,*988*	*N = 217*,*708*	*N = 215*,*975*	*N = 209*,*006*	*N = 201*,*728*	*N = 200*,*079*
Age						
< 20	5.6	5.1***	5.1***	4.8***	4.7***	4.6***
20–24	19.0	18.6**	18.4***	18.3***	17.8***	18.1***
25–29	28.2	27.7**	27.1***	26.8***	26.3***	26.3***
30–34	26.6	27.3***	27.2***	27.5***	28.0***	27.5***
35–39	16.2	16.6***	17.2***	17.5***	17.9***	18.1***
40+	4.5	4.7***	5.0***	5.2***	5.4***	5.4***
Formal Schooling						
< High School	45.2	43.6	41.5***	39.5***	38.1***	37.1***
High School	29.1	29.8***	30.7***	31.1***	30.9***	31.5***
Some College	15.9	16.3**	16.8***	17.4***	17.9***	17.8***
College+	9.8	10.4***	11.1***	12.1***	13.1***	13.6***
% First Birth	28.0	28.0	28.3*	28.5***	29.0***	29.3***
**Foreign-Born Mexican** *N = 691*,*670*	*N = 130*,*130*	*N = 127*,*779*	*N = 121*,*632*	*N = 111*,*796*	*N = 104*,*076*	*N = 96*,*257*
Age						
< 20	5.7	5.0***	4.8***	4.4***	4.0***	3.4***
20–24	19.1	18.8*	18.2***	17.9***	17.3***	17.0***
25–29	28.6	28.0**	27.3***	26.7***	26.1***	26.3***
30–34	26.2	27.2***	27.7***	28.0***	28.7***	28.4***
35–39	15.9	16.3***	17.0***	17.5***	18.2***	19.0***
40+	4.5	4.8**	5.2***	5.6***	5.8***	5.9***
Formal Schooling						
< High School	49.8	47.9***	45.2***	42.8***	40.8***	38.2***
High School	31.0	31.8***	33.2***	34.3***	34.6***	36.0***
Some College	13.1	13.8***	14.5***	15.3***	16.1***	16.5***
College+	6.1	6.5***	7.1***	7.7***	8.4***	9.2***
% First Birth	24.3	24.0	23.9*	23.5***	23.8*	24.3
**Foreign-Born Central Amer.** *N = 239*,*682*	*N = 33*,*556*	*N = 38*,*318*	*N = 39*,*606*	*N = 40*,*455*	*N = 40*,*710*	*N = 47*,*037*
Age						
< 20	6.3	6.6	7.3***	7.1***	7.7***	8.2***
20–24	19.4	18.9	19.9	20.5***	20.5***	22.5***
25–29	28.6	27.8*	26.5***	26.5***	25.8***	25.8***
30–34	26.9	26.9	25.6***	25.6***	25.4***	23.8***
35–39	15.0	15.8**	16.5***	16.2***	16.1***	15.3
40+	3.8	4.0	4.2**	4.1**	4.5***	4.4***
Formal Schooling						
< High School	58.3	59.3*	58.9	59.0	58.9	59.3**
High School	23.3	23.5	24.0*	23.9	23.8	24.3**
Some College	11.9	11.0***	11.0***	10.8***	10.7***	10.2***
College+	6.4	6.1	6.1	6.5	6.6	6.2
% First Birth	28.4	27.3**	27.6*	27.4**	27.3**	27.6*
**Foreign-Born South Amer.** *N = 100*,*299*	*N = 14*,*168*	*N = 16*,*612*	*N = 17*,*249*	*N = 17*,*104*	*N = 17*,*634*	*N = 17*,*532*
Age						
< 20	2.3	2.3	1.9*	1.5***	1.4***	1.5***
20–24	10.7	10.7	10.3	10.4	9.4***	9.6**
25–29	23.1	23.5	23.8	23.5	22.9	22.6
30–34	32.4	32.4	32.4	32.8	33.4	33.0
35–39	24.6	24.0	24.3	24.6	25.3	25.1
40+	6.8	7.2	7.3	7.3	7.5*	8.2***
Formal Schooling						
< High School	12.4	13.1	13.2*	11.3**	10.1***	10.0***
High School	21.9	23.2**	22.7	21.6	21.9	21.6
Some College	29.1	28.1	27.7**	27.5**	26.9***	26.8***
College+	36.6	35.6	36.4	39.6***	41.2***	41.6***
% First Birth	42.3	40.9**	40.6**	42.2	42.7	42.5
**Foreign-Born Puerto Rican** *N = 55*,*282*	*N = 7*,*585*	*N = 8*,*634*	*N = 9*,*342*	*N = 9*,*674*	*N = 10*,*188*	*N = 9*,*859*
Age						
< 20	8.6	8.7	8.0	7.2**	6.8***	6.7***
20–24	28.1	27.6	28.5	28.8	28.2	27.3
25–29	26.7	27.1	27.5	28.1*	29.2***	30.2***
30–34	21.9	21.5	20.0**	20.2**	20.3*	21.4
35–39	12.0	12.0	12.7	12.5	12.1	11.4
40+	2.8	3.0	3.3	3.3	3.4*	3.0
Formal Schooling						
< High School	21.4	19.9*	18.9***	17.9***	16.2***	14.8***
High School	29.5	32.1***	33.3***	33.7***	33.3***	35.2***
Some College	30.9	29.2*	29.8	30.6	30.9	30.3
College+	18.3	18.9	18.1	17.9	19.6*	19.8*
% First Birth	34.9	35.8	36.0	36.0	37.4**	37.8***
**Foreign-Born Cuban** *N = 35*,*367*	*N = 4*,*863*	*N = 5*,*306*	*N = 5*,*788*	*N = 6*,*424*	*N = 6*,*423*	*N = 6*,*563*
Age						
< 20	3.4	2.9	2.9	2.6**	2.7*	2.1***
20–24	21.2	19.3*	18.2***	16.7***	16.3***	16.3***
25–29	32.8	34.2	33.2	35.5**	33.3	31.4
30–34	23.3	25.9**	28.1***	28.6***	30.7***	32.6***
35–39	15.0	13.5*	13.6*	12.6***	13.1**	14.4
40+	4.3	4.2	4.1	4.0	4.1	3.2**
Formal Schooling						
< High School	9.8	9.2	9.7	10.4	10.2	8.4**
High School	44.5	45.2	45.9	45.2	42.6*	42.2*
Some College	28.6	26.7*	25.2***	24.9***	25.5**	25.1**
College+	17.1	18.9**	19.2**	19.5**	21.8***	24.3***
% First Birth	49.5	50.0	51.1	50.2	51.2	48.4

Data: National Center for Health Statistics Restricted-Use Natality Files, 2014–2019.

^1^ Includes all mothers who identified as Latina and reported being born outside of the United States. The foreign-born national/regional origin subgroups are included in this category. There were 147,459 foreign-born mothers whose national origin could not be identified.

Notes: Stars indicate statistical significance of changes in value relative to comparison period

(*P≤.05

**P≤.01

***P≤.001) calculated with a two-sample test of proportions using Stata’s “prtest” command.

Percentages may not sum to 100 due to rounding.

Table 3 shows the descriptive statistics for the outcome variables. Overall, these results indicate changes over time in levels of LBW, PTB, and inadequate prenatal care utilization, as well as variation in these outcomes across maternal nativity and country/region of origin. The aggregate groups of US- and foreign-born Latina mothers saw significant increases in their levels of LBW and PTB across the post-Trump periods, in comparison to the pre-Trump period. US-born Latinas saw significant declines in their levels of inadequate prenatal care utilization over time, while foreign-born Latinas saw significant decreases and increases in this outcome. Among the disaggregated groups of foreign-born Latinas, levels of LBW and PTB increased significantly among mothers from Mexico, and mothers from Central America also saw some significant increases in their levels of LBW and PTB over time. By contrast, levels of LBW and PTB remained generally similar over time among mothers from South America, Puerto Rico, and Cuba. Changes over time in levels of inadequate prenatal care utilization varied across groups of foreign-born Latina mothers. Mothers from Mexico and Puerto Rico saw some significant declines in their levels of inadequate prenatal care during the post-Trump periods, while mothers from Central America, South America, and Cuba generally saw significant increases in this outcome over time.

**Table 3:** Percentages of low birth weight, preterm birth, and inadequate prenatal care among US resident mothers across study periods (July 1 to Dec 31 in years 2014–2019).

	2014 Pre-Trump Period 2014	2015 Post-Trump Period	2016 Post-Trump Period	2017 Post-Trump Period	2018 Post-Trump Period	2019 Post-Trump Period
**US-Born Latina**						
% Low Birth Weight	6.1	6.2*	6.3**	6.4***	6.4***	6.5***
% Pre-Term Birth	9.6	9.6	9.6	10.0***	10.1***	10.7***
% Inadequate Prenatal Care	17.2	16.6***	16.7***	16.6***	16.0***	16.1***
**Foreign-Born Latina** ^**1**^						
% Low Birth Weight	5.3	5.4	5.5***	5.6***	5.7***	5.7***
% Pre-Term Birth	9.7	9.7	10.0**	10.1***	10.4***	11.1***
% Inadequate Prenatal Care	20.9	20.6*	21.0	20.5*	20.4***	22.5***
**Foreign-Born Mexican**						
% Low Birth Weight	5.0	5.2*	5.3***	5.4***	5.5***	5.6***
% Pre-Term Birth	9.4	9.2	9.7**	9.7**	10.1***	11.1***
% Inadequate Prenatal Care	21.1	20.9	21.0	20.5***	20.1***	21.2
**Foreign-Born Cent. Amer.**						
% Low Birth Weight	5.6	5.7	5.9	5.9	6.1*	5.8
% Pre-Term Birth	11.1	11.0	11.5	11.6*	11.8**	12.4***
% Inadequate Prenatal Care	26.2	25.6*	27.0*	26.6	27.1**	32.1**
**Foreign-Born South Amer.**						
% Low Birth Weight	4.5	4.6	4.8	4.8	4.5	4.7
% Pre-Term Birth	8.6	9.0	8.8	8.5	8.9	9.0
% Inadequate Prenatal Care	14.2	15.1*	17.0***	16.1***	16.1***	16.5***
**Foreign-Born Puerto Rican**						
% Low Birth Weight	8.0	7.8	7.5	7.8	7.4	8.1
% Pre-Term Birth	11.6	11.7	10.8	11.4	10.7	11.4
% Inadequate Prenatal Care	14.5	14.6	15.5	15.4	14.1	13.2*
**Foreign-Born Cuban**						
% Low Birth Weight	5.4	4.7	5.3	4.9	5.3	5.4
% Pre-Term Birth	11.2	10.5	10.3	10.0*	10.2	10.1
% Inadequate Prenatal Care	9.8	10.7	11.4**	11.0*	10.6	12.4***

Data: National Center for Health Statistics Restricted-Use Natality Files, 2014–2019.

^1^ Includes all mothers who identified as Latina and reported being born outside of the United States. The foreign-born national/regional origin subgroups are included in this category. There were 147,459 foreign-born mothers whose national origin could not be identified.

Notes: Stars indicate statistical significance of changes in value relative to comparison period

(*P≤.05

**P≤.01

***P≤.001) calculated with a two-sample test of proportions using Stata’s “prtest” command.

Percentages may not sum to 100 due to rounding.

### Birth outcomes during the Trump era

**Fig 1:**
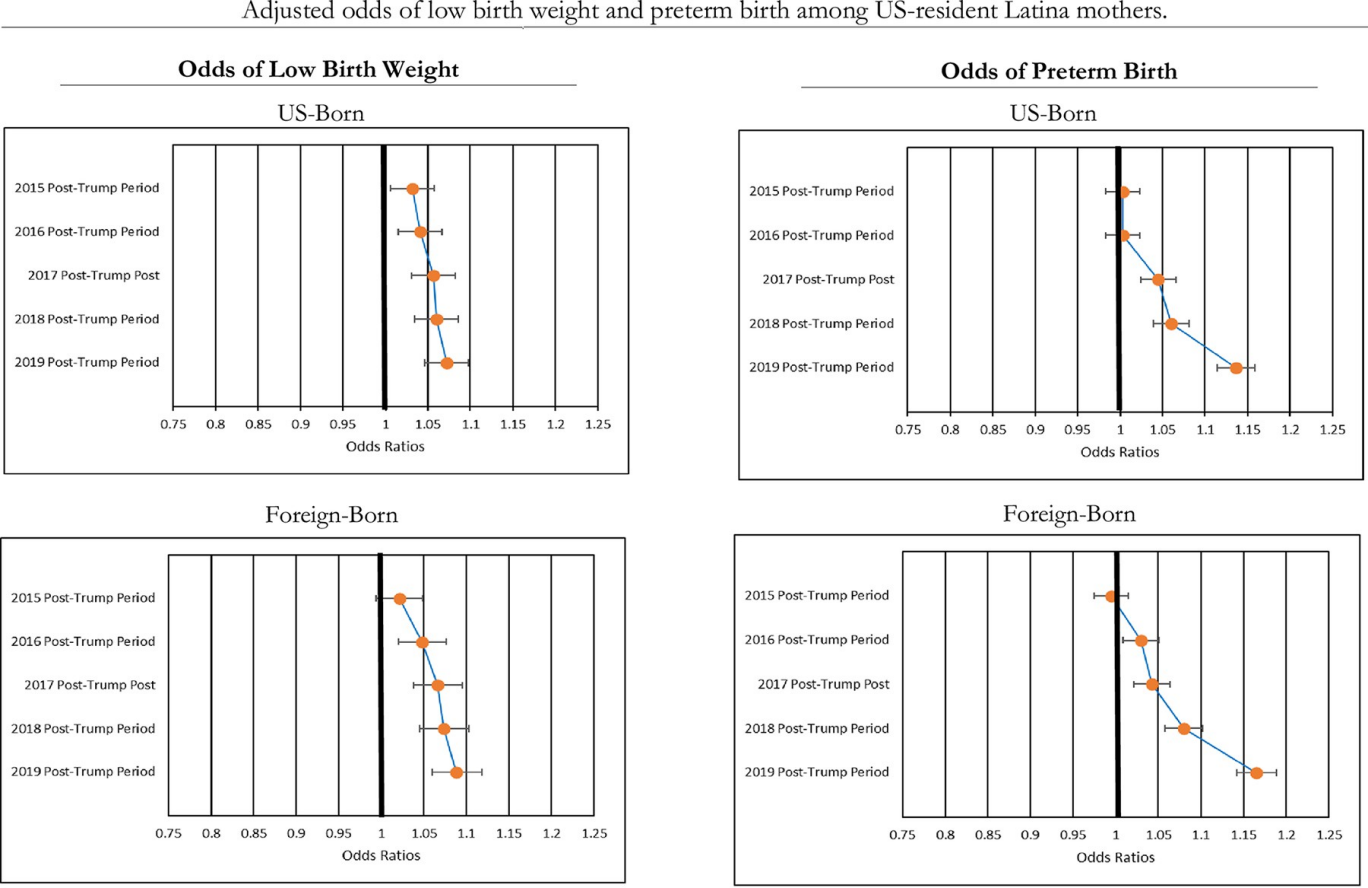
Adjusted odds of low birth weight and preterm birth, relative to pre-trump period by maternal nativity.

Fig 1 illustrates the results of the logistic regression models estimating the adjusted odds ratios (ORs) of LBW and PTB across the post-Trump study periods (2015–2019) compared with the pre-Trump period (2014) for the aggregate groups of US- and foreign-born Latina mothers. The 95% confidence intervals (CI) are also shown. Confirming the descriptive results in Table 3, these findings show that birth outcomes worsened significantly over time coincidental with Trump’s political career. In general, levels of LBW and PTB grew successively across each of the post-Trump periods among both US- and foreign-born Latina mothers, with the greatest increases occurring in the 2019 post-Trump period. From the 2014 pre-Trump period to the 2019 post-Trump period, US-born Latina mothers saw a 7% increase in the odds of LBW (OR = 1.07, 95% CI = 1.05–1.10, p < .000) and a 14% increase in the odds of PTB (OR = 1.14, 95% CI = 1.11–1.16, p < .000). For foreign-born Latina mothers, the odds of LBW and PTB increased by 9% (OR = 1.09, 95% CI = 1.06–1.12, p < .000) and 17% (OR = 1.17, 95% CI = 1.14–1.19, p < .000), respectively.

**Fig 2:**
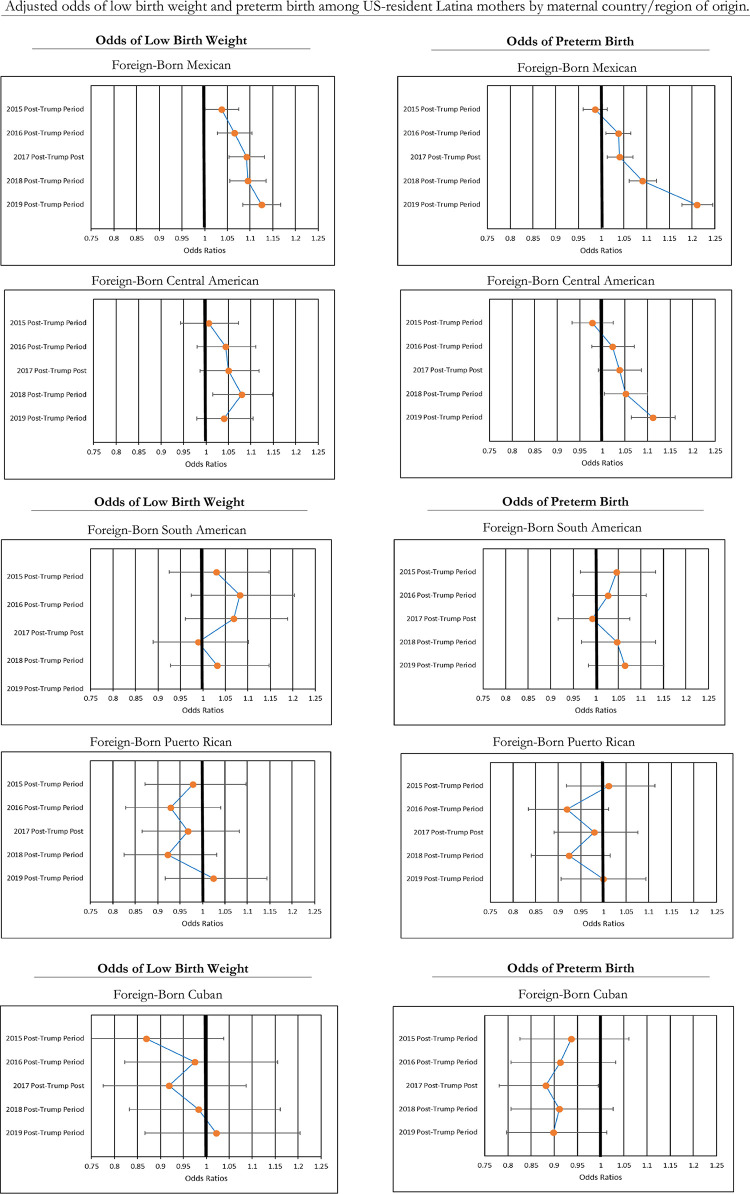
Adjusted odds of low birth weight and preterm birth, relative to pre-trump period by maternal country/region of origin.

Fig 2 shows how changes in birth outcomes among foreign-born Latina mothers varied by country/region of origin. According to these results, and consistent with the descriptive findings in Table 3, mothers from Mexico and Central America saw significant increases in LBW and PTB during the post-Trump study periods in comparison with the pre-Trump period, while mothers from South America, Puerto Rico, and Cuba saw no significant changes in these outcomes. Among mothers from Mexico, the odds of LBW and PTB generally grew successively over time, with the largest increase occurring in the 2019 post-Trump period. In comparison to the 2014 pre-Trump period, their odds of LBW increased by 12% (OR = 1.12, 95% CI = 1.08–1.17, p < .000) and their odds of PTB increased by 21% (OR = 1.21, 95% CI = 1.18–1.24, p < .000) during the 2019 post-Trump period. Mothers from Central America saw a significant increase in their odds of LBW during the 2018 post-Trump period (OR = 1.08, 95% CI = 1.01–1.15, p < .05), and significant increases in their odds of PTB in the 2018 (OR = 1.05, 95% CI = 1.00–1.10, p < .05) and 2019 (OR = 1.11, 95% CI = 1.06–1.16, p < .000) post-Trump periods.

### Prenatal care utilization during the Trump era

**Fig 3:**
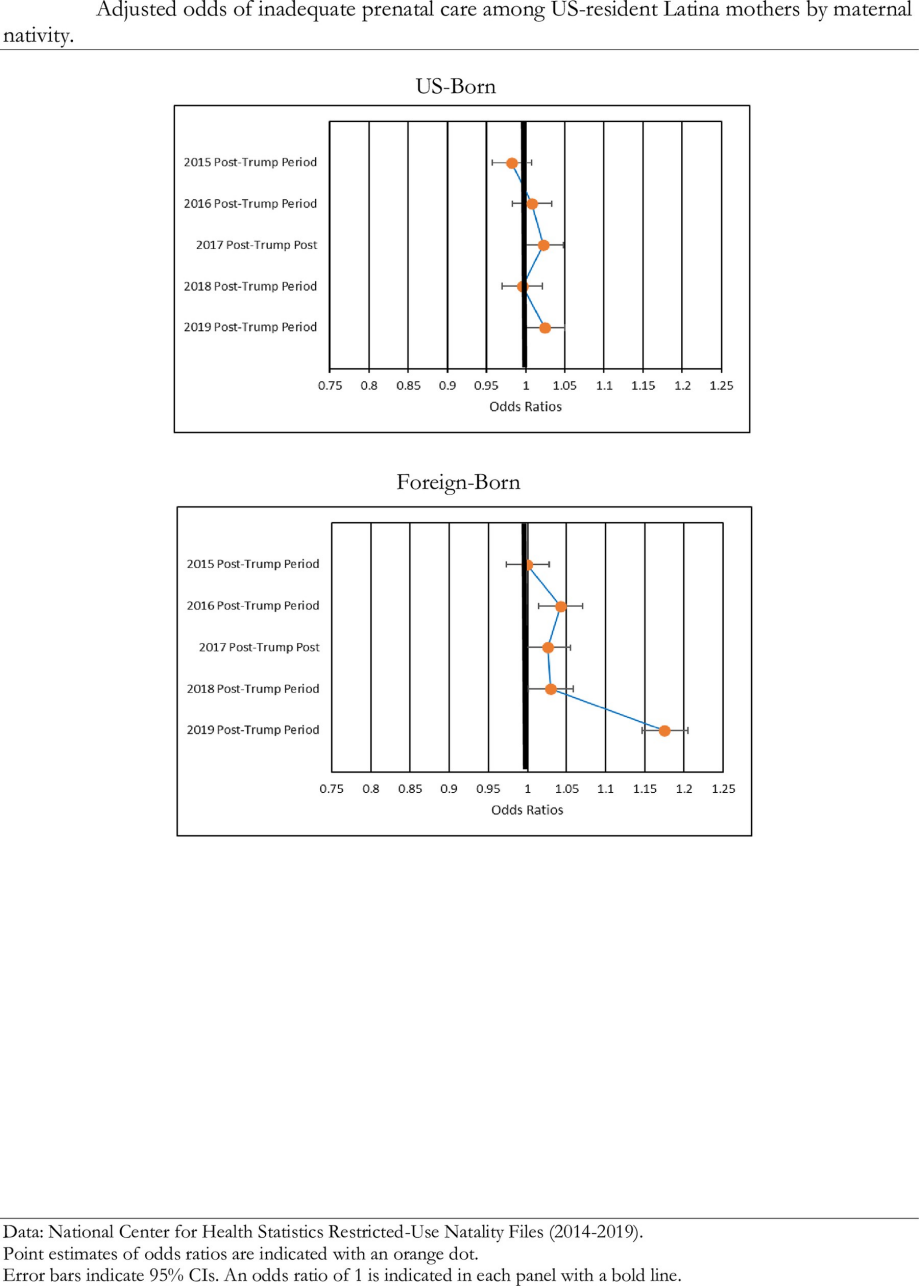
Adjusted odds of inadequate prenatal care relative to pre-trump period by maternal nativity.

Fig 3 provides the results of the logistic regression models with inadequate prenatal care utilization as the outcome for the aggregate groups of US- and foreign-born Latina mothers. According to these results, US-born Latina mothers saw significant increases in their odds of using inadequate levels of prenatal care in the 2017 (OR = 1.02, 95% CI = 1.01–1.04, p < .01) and the 2019 (OR = 1.02, 95% CI = 1.01–1.04, p < .01) post-Trump periods. Foreign-born Latina mothers saw significant increases in their odds of using inadequate prenatal care during the 2016, 2017, 2018, and 2019 post-Trump periods, with their most pronounced increase occurring in the 2019 post-Trump period. In comparison to the 2014 pre-Trump period, foreign-born Latina mothers’ odds of using inadequate levels of prenatal care in the 2019 post-Trump period increased by 17% (OR = 1.17, 95% CI = 1.16–1.19, p < .000).

**Fig 4:**
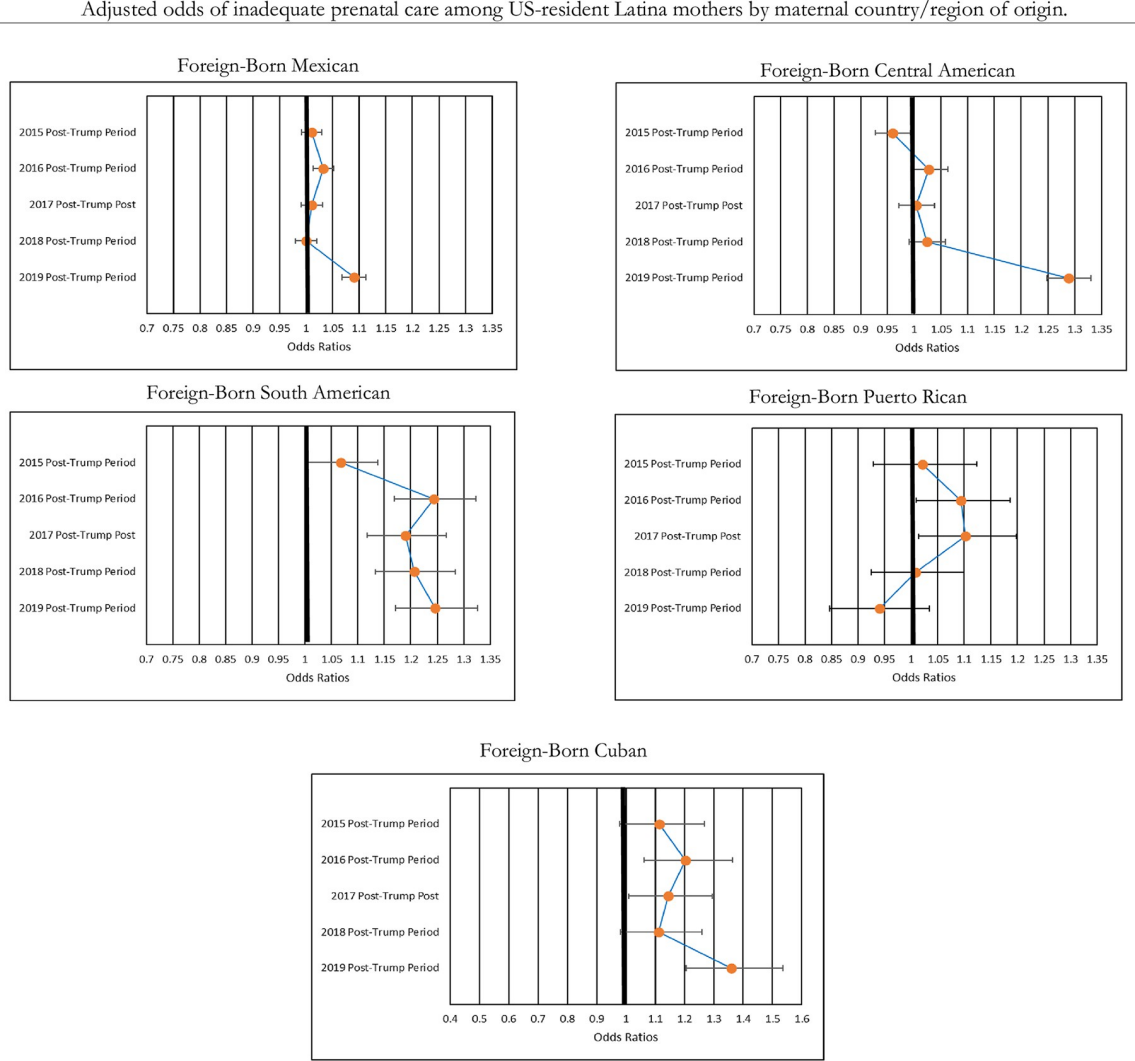
Adjusted odds of inadequate prenatal care, relative to pre-trump period by maternal country/region of origin.

Fig 4 shows how changes in inadequate prenatal care among foreign-born Latina mothers varied by country/region of origin. Overall, and consistent with the descriptive findings in Table 3, these results do not generally reflect expected trends based on the observed changes in birth outcomes. In other words, groups who saw significant increases in their levels of LBW and PTB did not see similarly consistent increases in their levels of inadequate prenatal care. Mothers from Mexico saw significant increases in their odds of using inadequate levels of prenatal care during only the 2016 and 2019 post-Trump periods, and mothers from Central America saw a significant increase in their odds of using inadequate levels of prenatal care during only the 2019 post-Trump period. Notably, Central American mothers’ odds of using inadequate levels of prenatal care increased by 29% (OR = 1.29, 95% CI = 1.25–1.33, p < .000) during the 2019 post-Trump period in comparison to the 2014 pre-Trump period.

Despite having no significant changes in their levels of LBW and PTB, mothers from South America, Puerto Rico, and Cuba saw some significant increases in their levels of inadequate prenatal care utilization. Among mothers from South America, the odds of using inadequate levels of prenatal care increased significantly during the 2016, 2017, 2018, and 2019 post-Trump periods, with their largest increase occurring in 2019 (OR = 1.25, 95% CI = 1.17–1.33, p < .000). Mothers from Puerto Rico saw significant increases in their odds of using inadequate levels of prenatal care in the 2015 and 2016 post-Trump study periods, and mothers from Cuba saw significant increases in their odds of using inadequate levels of prenatal care during the 2016, 2017, and 2019 post-Trump study periods (see Fig 4). Similar to Central American mothers, mothers from Cuba saw substantially large increases in their odds of using inadequate levels of prenatal care in the 2019 post-Trump period. For Cuban mothers, their odds of using inadequate levels of prenatal care increased by 36% (OR = 1.36, 95% CI = 1.21–1.54, p < .000) during the 2019 post-Trump period in comparison to the 2014 pre-Trump period.

### Sensitivity analyses

Our sensitivity testing produced essentially the same results as the primary analyses. Results of our imputation models controlling for maternal marital status in addition to maternal age, formal schooling, and parity are available upon request.

## Discussion

The Latinx population in the United States was targeted by racist and xenophobic rhetoric and policy during the entirety of Trump’s political career, from his presidential campaign through his administration, and people from Mexico and Central America were particularly threatened [1–7]. The sociopolitical environment during the Trump era may have therefore represented a significant source of chronic stress among the Latinx population, and there are important reasons to expect that these experiences of stress varied by nativity and nationality. Sociopolitical stressors during pregnancy have been linked to adverse birth outcomes, and previous research has linked the discrete event of Trump’s presidential election to negative changes in birth outcomes among the aggregate population of Latina mothers [13]. No study to date, however, has investigated whether birth outcomes among Latina mothers have changed over the entire course of Trump’s political career and how outcomes have varied by nativity and country/region of origin.

Addressing this important gap in the literature, we examined temporal associations between the Trump sociopolitical environment and birth outcomes among Latina mothers over a six-year period and investigated potential heterogeneity in outcomes by nativity and country/region of origin. Our results indicate that both US- and foreign-born Latina mothers experienced increasingly higher risks of adverse birth outcomes over the course of Trump’s political career from 2015 to 2019. For US-born Latina mothers, the odds of delivering a low birthweight infant increased by 3.1–7.2% and the odds of delivering a preterm infant increased by 0.1–13.7% across the observed years of Trump’s political career (2015, 2016, 2017, 2018, 2019), in comparison to a pre-Trump period (2014). These changes in birth outcomes suggest that there were approximately 3,700 more low birthweight infants and approximately 5,300 more preterm infants delivered by US-born mothers during the Trump era, compared to the counterfactual scenario in which the pre-Trump levels of LBW and PTB had continued. Among foreign-born Latina mothers, the odds of delivering a low birthweight infant increased by 2.1–8.9% and the odds of delivering a preterm infant increased by 1.0–16.5% during 2015–2019. These results imply that the population of immigrant Latina mothers in the US delivered approximately 3,300 more low birthweight infants and nearly 6,300 more preterm infants during the Trump era than what would have been expected based on the pre-Trump data.

In our analysis examining heterogeneity in birth outcomes among immigrant Latina mothers, we find notable variation across nativity and nationality. Consistent with our hypotheses, our results indicate that the Trump sociopolitical environment was associated with significant increases in the risk of adverse birth outcomes among foreign-born Mexican and Central American mothers, but not among mothers from South America, Puerto Rico, and Cuba. These findings may reflect important differences in stress and anxiety among Latina mothers due to variations in migration experiences, citizenship status, and racism experienced by people from different Latin American countries. People from Mexico and Central America may have experienced greater levels of stress and anxiety because they are more likely to be undocumented, to have undocumented family members, or to be suspected as being undocumented [29], and they were disproportionately targeted by Trump’s racist rhetoric and policies [3–9]. People from Puerto Rico are US citizens and people from South America and Cuba have generally experienced preferential migration treatment from the US government, allowing them to have higher levels of legal residency and citizenship [42,52]. Further, these groups have not been as explicitly targeted by Trump during his political career. Thus, mothers from South America, Puerto Rico, and Cuba may not have experienced as much added stress in the Trump sociopolitical environment.

Increases in adverse birth outcomes were most severe among foreign-born Mexican mothers. For this group, the odds of delivering a low birthweight infant increased by 3.7–12.5% and the odds of delivering a preterm infant increased by 1.0–21.1% across 2015–2019, implying that immigrant mothers from Mexico delivered approximately 2,300 more low birthweight infants and roughly 3,800 more preterm infants during the Trump era, in comparison to what would have been expected based on the pre-Trump data. Together with the results among the aggregate population of foreign-born Latina mothers, these findings suggest that low birthweight and preterm deliveries were disproportionately concentrated among foreign-born Mexican mothers during the years associated with Trump’s political career. Despite making up about 55% of all births to foreign-born Latina mothers during the study period (2014–2019; see Table 2), foreign-born Mexican mothers experienced approximately 70% of the excess low birthweight deliveries and 60% of the excess preterm birth deliveries that occurred during the observed Trump era (2015–2019).

In addition to examining birth outcomes, we also investigated whether and how the use of prenatal care among Latina mothers changed over time across the years associated with Trump’s political career. Past research has found that the enactment and enforcement of harsh immigration policies have been associated with significant reductions in the use of prenatal care among expectant Latina mothers [22,23,47], and one recent study found significant declines in prenatal care visits among expectant immigrant Latina mothers from Mexico and Central America in Houston, Texas following the start of Trump’s presidential campaign in the summer of 2015 [21]. In contrast to these studies, our results suggest that targeted groups of Latina mothers did not generally experience worse levels of prenatal care utilization over the course of Trump’s political career.

Given our findings on adverse birth outcomes, we expected to observe significant increases in levels of inadequate prenatal care utilization among the aggregate groups of US- and foreign-born Latina mothers as well as among the subgroups of immigrant Latina mothers from Mexico and Central America. Among these groups, however, our results show that increases in inadequate prenatal care occurred at relatively low levels and only during a few of the post-Trump study periods. Only mothers from Central America saw a very substantial increase (29%) in their levels of inadequate prenatal care during the 2019 post-Trump period. We find that immigrant mothers from South America, Puerto Rico, and Cuba also saw significant increases in their levels of inadequate prenatal care, although they experienced no significant increases in their levels of LBW and PTB. These worsening trends in prenatal care among South American, Puerto Rican, and Cuban mothers were not associated with observable changes in the data, such as changes over time in demographic composition or in the geography of where births took place. Future research should investigate these surprising and troubling increases in inadequate prenatal care, more deeply.

Taken together, the unexpected findings on prenatal care suggest that the worsening of adverse birth outcomes among Mexican and Central American mothers are not generally explained by a co-occurring decline in prenatal care. However, because our results illustrate patterns at the national level, more research is needed on the extent to which changes in the use of prenatal care may have varied geographically. Given previous research that found significant declines in prenatal visits among expectant immigrant Latina mothers from Mexico and Central America in Houston, Texas following the initiation of Trump’s presidential campaign [21], there are important reasons to expect that certain geographic contexts may have experienced unique changes in health care during Trump’s political career. Further, as our measure only captures the quantity and timing of prenatal care, more research is also needed to understand whether and how the quality of prenatal care has changed and potentially affected birth outcomes among Latina mothers during the Trump era.

## Limitations

Despite the strengths of this study, we acknowledge several limitations. First, although we controlled for important maternal factors associated with birthweight, it remains possible that there are unmeasured and unobserved confounding variables that were not considered in our analysis. Further, although existing studies show that Latinx people have experienced increased stress and anxiety due the ways that Trump has influenced the sociopolitical environment [3–9], our assumption that mothers in our sample experienced increased stress because of Trump may be flawed. Potential increases in stress among Latina mothers during the study period may have been caused by other factors coincidence with Trump’s political career, such as the enforcement of immigration policies that were in place before Trump was elected. Accordingly, we present our findings as temporal associations and make no statements regarding causality between the Trump treatment periods and infant and maternal health outcomes.

Second, our results may be subject to selection biases from changes in the composition of mothers giving birth in the US. Declines in the number of births among more advantaged and healthier mothers would cause us to overestimate the potential true effect of the Trump treatment periods on birth and prenatal care outcomes. Our analysis of maternal sociodemographic characteristics over time, however, shows that traditional risk factors associated with LBW, PTB, and inadequate use of prenatal care were generally less prevalent in the post-Trump periods compared with the pre-Trump period.

Third, our analysis examining temporal changes in birth and prenatal care outcomes over time uses one year of data for the pre-Trump study period. Using more pre-Trump years extending into the past can be important to evaluate and rule-out pre-treatment trends as a potential source of confounding. In our early analyses for this study, we examined birth and prenatal care outcomes using the restricted-use birth records from as far back as 2010 and found that trends in these outcomes were generally stable or declining prior to 2015 (2010–2014). Accordingly, the 2010–2019 data generated results that were statistically and substantively identical to those presented here. We opted to restrict the analysis to the 2014–2019 data, however, for two reasons. First, because the results from the 2014–2019 data were more conservative, given that they do not account for some declines in the outcomes prior to 2014. Second, and most importantly, because some measures used in our analysis were only available for certain states prior to 2014. In 2003, the NCHS adopted a revision to birth certificates that changed the standard reporting of information in the natality data, and these revisions were implemented across states over time. In 2014, all states fully transitioned to the revised birth certificate for the measures used in our analysis. Thus, we considered the data prior to 2014 as incomplete and found no statistical or substantive value in adding these years to our analysis.

Fourth, the analysis excludes information on maternal health status, which may have important implications for our observed results. The NCHS birth records data includes a select few measures on maternal health, including pre-pregnancy diabetes, gestational diabetes, pre-pregnancy hypertension, gestational hypertension, and eclampsia, which are all strongly associated with adverse birth outcomes [53]. In additional sensitivity analyses (not shown) we included these variables in our original models and the results were generally identical to the reported results, but the effect sizes were diminished in some cases. However, we did not include these variables in our final analysis because we considered them endogenous to the Trump periods, due to their shared relationship with stress [54]. Future research should consider how adverse birth outcomes are shaped by both health conditions associated with stress and stressful sociopolitical contexts.

Fifth, the restricted data files we used enabled us to identify foreign-born Latina mothers by country/region of origin but lacked information that would allow us to identify US-born Latina mothers across nationalities. Understanding temporal changes in birth and prenatal care outcomes among these groups of mothers is important because Trump’s attacks against immigrants from Latin American countries create negative health implications that extend beyond intended targets, affecting US-born co-ethnic groups.

Lastly, we could not identify the mechanisms that account for the temporal relationships between the Trump study periods and the observed birth outcomes. Although potentially flawed (as mentioned above), we considered Trump’s political career as an environmental stressor for expectant Latina mothers, given existing evidence indicating that Latinx people have experienced increased stress and anxiety due to Trump’s sociopolitical influence [3–9]. Environmental stressors can affect birth outcomes through several pathways, including the mother’s biological and behavioral responses to stress that could harm the developing fetus and result in adverse birth outcomes among infants. We investigated prenatal care utilization as a potential pathway but found that changes in prenatal care did not generally explain the observed worsening of birth outcomes among Latina mothers during the Trump study periods. Still, several other potential pathways could account for the temporal changes in birth outcomes that occurred during the Trump study periods, including maternal changes in diet and exercise, family income and social support, and quality of health care. Future research should examine these and other potential pathways to uncover more information about the temporal linkages between the Trump sociopolitical era and adverse birth outcomes among Latina mothers.

## Conclusion

Our findings add important and timely updates to the growing literature on the temporal linkages between the Trump sociopolitical environment and adverse birth outcomes among Latina mothers in the US [13,14]. In their recent study examining changes in birth outcomes among the aggregate population of Latina mothers, Gemmill and colleagues find that the risk of PTB increased by 3.2–3.6% in the year following Trump’s 2016 presidential election [13]. Our results show that the risks of adverse birth outcomes continued to increase in the subsequent years of Trump’s presidential term, and that the worsening of birth outcomes were most severe among foreign-born Mexican mothers. By extending the period of investigation to account for Trump’s entire political career, and by analyzing births across nativity and country/region of origin, our findings draw attention to the possibility that the Trump era may have represented a source of chronic stress among Latina mothers, and provide new evidence on how racism in the sociopolitical environment may vary across subgroups within the Latinx population. Findings from this study may therefore be considered in the creation of targeted interventions to improve birth outcomes among expectant Latina mothers in general, and specifically among those from Mexico and Central America. Indeed, as Latina women are distinct patients who experience different protective and risk factors to their health and the health of their children due to differences in their family’s country or origin and whether they identify as immigrants [55], it is critical that health care providers establish a greater understanding of the varying health needs within the often-aggregated group of Latina women.

A growing body of literature emphasizes the embodied stress that Latinx people in the US experienced during Trump’s political career, due to his racist and anti-immigrant rhetoric and policy [1–9]. Building off existing work in this area, we analyzed restricted-use natality files to provide the first nationwide study of changes in birth and prenatal care outcomes during the Trump era among Latina mothers. Net of changes in maternal sociodemographic characteristics, our results suggest that periods following Trump’s presidential campaign, election, and administration were associated with particularly sizeable increases in low birthweight and preterm birth among infants born in the US to immigrant mothers from Mexico.

## Supporting information

S1 File (DOCX)
