# Supplementary material for: Birth and prenatal care outcomes of Latina mothers in the Trump era: Analysis by nativity and country/region of origin
Source: PLoS One. 2023 Mar 1;18(3):e0281803. doi: 10.1371/journal.pone.0281803 (PMC9977052; doi:10.1371/journal.pone.0281803)
Supplement: S1 File — (DOCX) [file pone.0281803.s001.docx]

**Birth and Prenatal Care Outcomes of Latina Mothers in the Trump Era: Analysis by Nativity and Country/Region of Origin**

Carmen Gutierrez^1*^, Nathan T. Dollar^2^

^1^Department of Public Policy, Carolina Population Center, University of North Carolina at Chapel Hill

^2^, Carolina Population Center, University of North Carolina at Chapel Hill

^*^**Corresponding Author**

Email: [carmen.gutierrez@unc.edu](mailto:carmen.gutierrez@unc.edu)

**SUPPORTING INFORMATION**

**Section 1. Multivariable logistic regression equations used to identify the temporal associations between exposure to the Trump treatment periods and each outcome of interest: LBW, PTB, and inadequate prenatal care utilization.**

Equation 1, LBW:

$\log\left( \frac{lbw}{1-lbw} \right)=\beta_{0}+ \beta_{1_{i}}{age}_{i}+ \beta_{2_{j}}{education}_{j}+ \beta_{3}parity+ \beta_{4_{k}}{period}_{k}$

Equation 2, PTB:

$\log\left( \frac{PTB}{1-PTB} \right)=\beta_{0}+ \beta_{1_{i}}{age}_{i}+ \beta_{2_{j}}{education}_{j}+ \beta_{3}parity+ \beta_{4_{k}}{period}_{k}$

Equation 3, inadequate prenatal care utilization:

$$\log\left( \frac{inadequate}{1-inadequate} \right)=\beta_{0}+ \beta_{1_{i}}{age}_{i}+ \beta_{2_{j}}{education}_{j}+ \beta_{3}parity+ \beta_{4_{k}}{period}_{k}$$

**S1 Table. Adjusted multivariable logistic regression models for the associations between the Trump study periods and low birthweight among Latina mothers, 2014-2019.**

|  |  |  |  |  |  |  |  |  |  |
| --- | --- | --- | --- | --- | --- | --- | --- | --- | --- |
| **US-born Latinas** | |  |  |  |  |  |  | |  |
|  |  |  | OR |  | P-value |  | 95% CI | |  |
|  | Trump periods |  |  |  |  |  |  |  |  |
|  | 2015 |  | 1.031375 |  | 0.014 |  | 1.006336 | 1.057037 |  |
|  | 2016 |  | 1.040593 |  | 0.001 |  | 1.015457 | 1.066351 |  |
|  | 2017 |  | 1.056181 |  | 0.000 |  | 1.030751 | 1.082239 |  |
|  | 2018 |  | 1.059761 |  | 0.000 |  | 1.03426 | 1.085892 |  |
|  | 2019 |  | 1.071633 |  | 0.000 |  | 1.045994 | 1.0979 |  |
|  |  |  |  |  |  |  |  |  |  |
|  |  |  |  |  |  |  |  |  |  |
| **Foreign-born Latinas** | |  |  |  |  |  |  |  |  |
|  |  |  | OR |  | P-value |  | 95% CI | |  |
|  | Trump periods |  |  |  |  |  |  |  |  |
|  | 2015 |  | 1.021342 |  | 0.122 |  | 0.994357 | 1.049059 |  |
|  | 2016 |  | 1.048084 |  | 0.001 |  | 1.020499 | 1.076414 |  |
|  | 2017 |  | 1.066259 |  | 0.000 |  | 1.038065 | 1.095219 |  |
|  | 2018 |  | 1.07347 |  | 0.000 |  | 1.044881 | 1.102842 |  |
|  | 2019 |  | 1.088499 |  | 0.000 |  | 1.05953 | 1.118259 |  |
|  |  |  |  |  |  |  |  |  |  |
|  |  |  |  |  |  |  |  |  |  |
| **Foreign-born Mexicans** | |  |  |  |  |  |  |  |  |
|  |  |  | OR |  | P-value |  | 95% CI | |  |
|  | Trump periods |  |  |  |  |  |  |  |  |
|  | 2015 |  | 1.037298 |  | 0.042 |  | 1.001389 | 1.074495 |  |
|  | 2016 |  | 1.065331 |  | 0.000 |  | 1.028221 | 1.103781 |  |
|  | 2017 |  | 1.091911 |  | 0.000 |  | 1.053241 | 1.132 |  |
|  | 2018 |  | 1.094809 |  | 0.000 |  | 1.055331 | 1.135764 |  |
|  | 2019 |  | 1.124855 |  | 0.000 |  | 1.083681 | 1.167593 |  |
|  |  |  |  |  |  |  |  |  |  |
|  |  |  |  |  |  |  |  |  |  |
| **Foreign-born Central Americans** | | |  |  |  |  |  |  |  |
|  |  |  | OR |  | P-value |  | 95% CI | |  |
|  | Trump periods |  |  |  |  |  |  |  |  |
|  | 2015 |  | 1.005837 |  | 0.858 |  | 0.943856 | 1.071888 |  |
|  | 2016 |  | 1.043362 |  | 0.184 |  | 0.98003 | 1.110786 |  |
|  | 2017 |  | 1.050069 |  | 0.124 |  | 0.986649 | 1.117566 |  |
|  | 2018 |  | 1.079526 |  | 0.015 |  | 1.014753 | 1.148432 |  |
|  | 2019 |  | 1.039986 |  | 0.204 |  | 0.978914 | 1.104868 |  |
|  |  |  |  |  |  |  |  |  |  |
|  |  |  |  |  |  |  |  |  |  |
| **Foreign-born South Americans** | | |  |  |  |  |  |  |  |
|  |  |  | OR |  | P-value |  | 95% CI | |  |
|  | Trump periods |  |  |  |  |  |  |  |  |
|  | 2015 |  | 1.029994 |  | 0.590 |  | 0.924901 | 1.147029 |  |
|  | 2016 |  | 1.082353 |  | 0.142 |  | 0.973786 | 1.203024 |  |
|  | 2017 |  | 1.068611 |  | 0.220 |  | 0.96099 | 1.188283 |  |
|  | 2018 |  | 0.989783 |  | 0.851 |  | 0.889363 | 1.101542 |  |
|  | 2019 |  | 1.031579 |  | 0.566 |  | 0.927633 | 1.147172 |  |
|  |  |  |  |  |  |  |  |  |  |
|  |  |  |  |  |  |  |  |  |  |
| **Foreign-born Puerto Ricans** | | |  |  |  |  |  |  |  |
|  |  |  | OR |  | P-value |  | 95% CI | |  |
|  | Trump periods |  |  |  |  |  |  |  |  |
|  | 2015 |  | 0.978058 |  | 0.704 |  | 0.872178 | 1.096791 |  |
|  | 2016 |  | 0.928904 |  | 0.203 |  | 0.829149 | 1.04066 |  |
|  | 2017 |  | 0.967663 |  | 0.565 |  | 0.865204 | 1.082255 |  |
|  | 2018 |  | 0.922216 |  | 0.156 |  | 0.824665 | 1.031307 |  |
|  | 2019 |  | 1.024213 |  | 0.672 |  | 0.916941 | 1.144036 |  |
|  |  |  |  |  |  |  |  |  |  |
|  |  |  |  |  |  |  |  |  |  |
| **Foreign-born Cubans** | |  |  |  |  |  |  |  |  |
|  |  |  | OR |  | P-value |  | 95% CI | |  |
|  | Trump periods |  |  |  |  |  |  |  |  |
|  | 2015 |  | 0.868751 |  | 0.122 |  | 0.726917 | 1.038259 |  |
|  | 2016 |  | 0.975066 |  | 0.771 |  | 0.822618 | 1.155767 |  |
|  | 2017 |  | 0.918354 |  | 0.322 |  | 0.775948 | 1.086893 |  |
|  | 2018 |  | 0.983105 |  | 0.841 |  | 0.832563 | 1.160868 |  |
|  | 2019 |  | 1.021351 |  | 0.802 |  | 0.866102 | 1.204429 |  |
|  |  |  |  |  |  |  |  |  |  |
|  |  |  |  |  |  |  |  |  |  |

OR = odds ratio.

CI = confidence interval.

Note: All models are adjusted for maternal age, formal schooling, and parity.

**S2 Table. Adjusted multivariable logistic regression models for the associations between the Trump study periods and preterm birth among Latina mothers, 2014-2019.**

| **US-born Latinas** | |  |  |  |  |  |  |  |  |
| --- | --- | --- | --- | --- | --- | --- | --- | --- | --- |
|  |  |  | OR |  | P-value |  | 95% CI | |  |
|  | Trump periods |  |  |  |  |  |  |  |  |
|  | 2015 |  | 1.002855 |  | 0.28 |  | 0.98298 | 1.023133 |  |
|  | 2016 |  | 1.003146 |  | 0.31 |  | 0.983331 | 1.02336 |  |
|  | 2017 |  | 1.044641 |  | 4.32 |  | 1.024161 | 1.065531 |  |
|  | 2018 |  | 1.059698 |  | 5.75 |  | 1.038971 | 1.080839 |  |
|  | 2019 |  | 1.136448 |  | 12.89 |  | 1.114557 | 1.158769 |  |
|  |  |  |  |  |  |  |  |  |  |
|  |  |  |  |  |  |  |  |  |  |
| **Foreign-born Latinas** | |  |  |  |  |  |  |  |  |
|  |  |  | OR |  | P-value |  | 95% CI | |  |
|  | Trump periods |  |  |  |  |  |  |  |  |
|  | 2015 |  | 0.994516 |  | 0.596 |  | 0.974523 | 1.014919 |  |
|  | 2016 |  | 1.029498 |  | 0.005 |  | 1.008891 | 1.050526 |  |
|  | 2017 |  | 1.042177 |  | 0.000 |  | 1.021177 | 1.06361 |  |
|  | 2018 |  | 1.079627 |  | 0.000 |  | 1.057814 | 1.10189 |  |
|  | 2019 |  | 1.165317 |  | 0.000 |  | 1.142044 | 1.189063 |  |
|  |  |  |  |  |  |  |  |  |  |
|  |  |  |  |  |  |  |  |  |  |
| **Foreign-born Mexicans** | |  |  |  |  |  |  |  |  |
|  |  |  | OR |  | P-value |  | 95% CI | |  |
|  | Trump periods |  |  |  |  |  |  |  |  |
|  | 2015 |  | 0.986471 |  | 0.316 |  | 0.960545 | 1.013097 |  |
|  | 2016 |  | 1.037585 |  | 0.007 |  | 1.010247 | 1.065663 |  |
|  | 2017 |  | 1.041002 |  | 0.004 |  | 1.012969 | 1.069811 |  |
|  | 2018 |  | 1.091029 |  | 0.000 |  | 1.06135 | 1.121538 |  |
|  | 2019 |  | 1.211007 |  | 0.000 |  | 1.178029 | 1.244908 |  |
|  |  |  |  |  |  |  |  |  |  |
|  |  |  |  |  |  |  |  |  |  |
| **Foreign-born Central Americans** | | |  |  |  |  |  |  |  |
|  |  |  | OR |  | P-value |  | 95% CI | |  |
|  | Trump periods |  |  |  |  |  |  |  |  |
|  | 2015 |  | 0.977645 |  | 0.344 |  | 0.932919 | 1.024514 |  |
|  | 2016 |  | 1.022768 |  | 0.338 |  | 0.976719 | 1.070988 |  |
|  | 2017 |  | 1.037726 |  | 0.113 |  | 0.991315 | 1.08631 |  |
|  | 2018 |  | 1.051126 |  | 0.032 |  | 1.004299 | 1.100136 |  |
|  | 2019 |  | 1.111726 |  | 0.000 |  | 1.064025 | 1.161565 |  |
|  |  |  |  |  |  |  |  |  |  |
|  |  |  |  |  |  |  |  |  |  |
| **Foreign-born South Americans** | | |  |  |  |  |  |  |  |
|  |  |  | OR |  | P-value |  | 95% CI | |  |
|  | Trump periods |  |  |  |  |  |  |  |  |
|  | 2015 |  | 1.045914 |  | 0.268 |  | 0.965969 | 1.132474 |  |
|  | 2016 |  | 1.02703 |  | 0.509 |  | 0.948898 | 1.111596 |  |
|  | 2017 |  | 0.992327 |  | 0.850 |  | 0.916093 | 1.074904 |  |
|  | 2018 |  | 1.047144 |  | 0.251 |  | 0.967934 | 1.132838 |  |
|  | 2019 |  | 1.064356 |  | 0.119 |  | 0.984007 | 1.151266 |  |
|  |  |  |  |  |  |  |  |  |  |
|  |  |  |  |  |  |  |  |  |  |
| **Foreign-born Puerto Ricans** | | |  |  |  |  |  |  |  |
|  |  |  | OR |  | P-value |  | 95% CI | |  |
|  | Trump periods |  |  |  |  |  |  |  |  |
|  | 2015 |  | 1.011321 |  | 0.819 |  | 0.91853 | 1.113485 |  |
|  | 2016 |  | 0.918627 |  | 0.084 |  | 0.834429 | 1.011321 |  |
|  | 2017 |  | 0.979153 |  | 0.662 |  | 0.890964 | 1.076071 |  |
|  | 2018 |  | 0.923694 |  | 0.099 |  | 0.840456 | 1.015175 |  |
|  | 2019 |  | 0.995159 |  | 0.919 |  | 0.905805 | 1.093327 |  |
|  |  |  |  |  |  |  |  |  |  |
|  |  |  |  |  |  |  |  |  |  |
| **Foreign-born Cubans** | |  |  |  |  |  |  |  |  |
|  |  |  | OR |  | P-value |  | 95% CI | |  |
|  | Trump periods |  |  |  |  |  |  |  |  |
|  | 2015 |  | 0.935995 |  | 0.301 |  | 0.825706 | 1.061015 |  |
|  | 2016 |  | 0.912816 |  | 0.147 |  | 0.806899 | 1.032636 |  |
|  | 2017 |  | 0.881491 |  | 0.042 |  | 0.780785 | 0.995187 |  |
|  | 2018 |  | 0.910427 |  | 0.127 |  | 0.806947 | 1.027178 |  |
|  | 2019 |  | 0.898158 |  | 0.081 |  | 0.796179 | 1.013199 |  |
|  |  |  |  |  |  |  |  |  |  |
|  |  |  |  |  |  |  |  |  |  |

OR = odds ratio.

CI = confidence interval.

Note: All models are adjusted for maternal age, formal schooling, and parity.

**S3 Table. Adjusted multivariable logistic regression models for the associations between the Trump study periods and inadequate prenatal care utilization among Latina mothers, 2014-2019.**

| **US-born Latinas** | |  |  |  |  |  |  |  |  |
| --- | --- | --- | --- | --- | --- | --- | --- | --- | --- |
|  |  |  | OR |  | P-value |  | 95% CI | |  |
|  | Trump periods |  |  |  |  |  |  |  |  |
|  | 2015 |  | 0.982208 |  | 0.028 |  | 0.966603 | 0.998065 |  |
|  | 2016 |  | 1.007971 |  | 0.329 |  | 0.992033 | 1.024165 |  |
|  | 2017 |  | 1.022257 |  | 0.007 |  | 1.006082 | 1.038693 |  |
|  | 2018 |  | 0.995438 |  | 0.577 |  | 0.979572 | 1.011562 |  |
|  | 2019 |  | 1.024142 |  | 0.003 |  | 1.007901 | 1.040646 |  |
|  |  |  |  |  |  |  |  |  |  |
|  |  |  |  |  |  |  |  |  |  |
| **Foreign-born Latinas** | |  |  |  |  |  |  |  |  |
|  |  |  | OR |  | P-value |  | 95% CI | |  |
|  | Trump periods |  |  |  |  |  |  |  |  |
|  | 2015 |  | 0.999712 |  | 0.970 |  | 0.98483 | 1.014818 |  |
|  | 2016 |  | 1.042014 |  | 0.000 |  | 1.026524 | 1.057737 |  |
|  | 2017 |  | 1.026095 |  | 0.001 |  | 1.010648 | 1.041779 |  |
|  | 2018 |  | 1.029534 |  | 0.000 |  | 1.013867 | 1.045445 |  |
|  | 2019 |  | 1.175269 |  | 0.000 |  | 1.15765 | 1.193157 |  |
|  |  |  |  |  |  |  |  |  |  |
|  |  |  |  |  |  |  |  |  |  |
| **Foreign-born Mexicans** | |  |  |  |  |  |  |  |  |
|  |  |  | OR |  | P-value |  | 95% CI | |  |
|  | Trump periods |  |  |  |  |  |  |  |  |
|  | 2015 |  | 1.010172 |  | 0.300 |  | 0.991041 | 1.029673 |  |
|  | 2016 |  | 1.032394 |  | 0.001 |  | 1.012603 | 1.052572 |  |
|  | 2017 |  | 1.010177 |  | 0.319 |  | 0.990258 | 1.030497 |  |
|  | 2018 |  | 0.999934 |  | 0.995 |  | 0.979729 | 1.020555 |  |
|  | 2019 |  | 1.090277 |  | 0.000 |  | 1.067985 | 1.113035 |  |
|  |  |  |  |  |  |  |  |  |  |
|  |  |  |  |  |  |  |  |  |  |
| **Foreign-born Central Americans** | | |  |  |  |  |  |  |  |
|  |  |  | OR |  | P-value |  | 95% CI | |  |
|  | Trump periods |  |  |  |  |  |  |  |  |
|  | 2015 |  | 0.95976 |  | 0.018 |  | 0.927619 | 0.993013 |  |
|  | 2016 |  | 1.027245 |  | 0.116 |  | 0.993351 | 1.062297 |  |
|  | 2017 |  | 1.003796 |  | 0.824 |  | 0.970765 | 1.037951 |  |
|  | 2018 |  | 1.0237 |  | 0.169 |  | 0.99013 | 1.058409 |  |
|  | 2019 |  | 1.288151 |  | 0.000 |  | 1.247925 | 1.329673 |  |
|  |  |  |  |  |  |  |  |  |  |
|  |  |  |  |  |  |  |  |  |  |
| **Foreign-born South Americans** | | |  |  |  |  |  |  |  |
|  |  |  | OR |  | P-value |  | 95% CI | |  |
|  | Trump periods |  |  |  |  |  |  |  |  |
|  | 2015 |  | 1.067284 |  | 0.046 |  | 1.001035 | 1.137918 |  |
|  | 2016 |  | 1.243267 |  | 0.000 |  | 1.168211 | 1.323146 |  |
|  | 2017 |  | 1.190255 |  | 0.000 |  | 1.117671 | 1.267553 |  |
|  | 2018 |  | 1.206471 |  | 0.000 |  | 1.133312 | 1.284353 |  |
|  | 2019 |  | 1.246354 |  | 0.000 |  | 1.171007 | 1.32655 |  |
|  |  |  |  |  |  |  |  |  |  |
|  |  |  |  |  |  |  |  |  |  |
| **Foreign-born Puerto Ricans** | | |  |  |  |  |  |  |  |
|  |  |  | OR |  | P-value |  | 95% CI | |  |
|  | Trump periods |  |  |  |  |  |  |  |  |
|  | 2015 |  | 1.02127 |  | 0.641 |  | 0.934868 | 1.115656 |  |
|  | 2016 |  | 1.093743 |  | 0.041 |  | 1.003618 | 1.19196 |  |
|  | 2017 |  | 1.101765 |  | 0.026 |  | 1.011595 | 1.199972 |  |
|  | 2018 |  | 1.008631 |  | 0.844 |  | 0.9257 | 1.098992 |  |
|  | 2019 |  | 0.940691 |  | 0.171 |  | 0.861839 | 1.026757 |  |
|  |  |  |  |  |  |  |  |  |  |
|  |  |  |  |  |  |  |  |  |  |
| **Foreign-born Cubans** | |  |  |  |  |  |  |  |  |
|  |  |  | OR |  | P-value |  | 95% CI | |  |
|  | Trump periods |  |  |  |  |  |  |  |  |
|  | 2015 |  | 1.114525 |  | 0.101 |  | 0.979078 | 1.26871 |  |
|  | 2016 |  | 1.20409 |  | 0.004 |  | 1.062064 | 1.36511 |  |
|  | 2017 |  | 1.143475 |  | 0.034 |  | 1.010305 | 1.294199 |  |
|  | 2018 |  | 1.112011 |  | 0.095 |  | 0.981762 | 1.259541 |  |
|  | 2019 |  | 1.360611 |  | 0.000 |  | 1.205658 | 1.535478 |  |
|  |  |  |  |  |  |  |  |  |  |
|  |  |  |  |  |  |  |  |  |  |

OR = odds ratio.

CI = confidence interval.

Note: All models are adjusted for maternal age, formal schooling, and parity.
